# ZFX Controls the Self-Renewal of Human Embryonic Stem Cells

**DOI:** 10.1371/journal.pone.0042302

**Published:** 2012-08-03

**Authors:** Sivan Harel, Edmund Y. Tu, Stuart Weisberg, Manuel Esquilin, Stuart M. Chambers, Becky Liu, Christian T. Carson, Lorenz Studer, Boris Reizis, Mark J. Tomishima

**Affiliations:** 1 Department of Microbiology and Immunology, Columbia University Medical Center, New York, New York, United States of America; 2 Developmental Biology Program, Sloan-Kettering Institute, New York, New York, United States of America; 3 BD Biosciences, La Jolla, California, United States of America; University of California Merced, United States of America

## Abstract

Embryonic stem cells (ESCs) and induced pluripotent stem cells (iPSCs) offer great promise in regenerative medicine and disease modeling due to their unlimited self-renewal and broad differentiation capacity. There is evidence that the growth properties and critical signaling pathways differ between murine and human ESCs; therefore, it is essential to perform functional studies to test the putatively conserved mechanisms of pluripotent stem cell self-renewal between species. Previously, we identified the transcription factor Zfx as a key regulator of self-renewal in murine ESCs. Here we extend those findings to human ESCs. ZFX knockdown in hESCs hindered clonal growth and decreased colony size after serial replating. ZFX overexpression enhanced clone formation in the presence of Y-27632, increased colony size at low density and decreased expression of differentiation-related genes in human ESCs. ZFX-overexpressing hESCs resisted spontaneous differentiation but could be directed to differentiate into endodermal and neural cell fates when provided with the appropriate cues. Thus, ZFX acts as a molecular rheostat regulating the balance between self-renewal and differentiation in hESCs, revealing the close evolutionary conservation of the self-renewal mechanisms in murine and human ESCs.

## Introduction

Embryonic stem cells (ESCs) and the related induced pluripotent stem cells (iPSCs) are unique cells capable of giving rise to all tissues of the adult organism. These pluripotent stem cells (PSCs) can be exponentially expanded in culture while retaining their differentiation potential. The traits of pluripotency and continuous self-renewal underlie the value of PSCs as a potential source for cell replacement therapies and disease modeling, as well as a tool to study normal human development [1]–[3]. The pluripotency of both mouse and human ESCs is regulated by a network of ESC-specific transcription factors including Oct4, Nanog, Sox2 and their binding partners and targets [4], [5]. These factors promote the undifferentiated state by positively regulating expression of pluripotency related genes while repressing lineage-specific gene expression and maintaining the unique permissive chromatin structure of ESCs. In addition to ESC-specific transcription factors, additional sets of regulators appear essential for the self-renewal of undifferentiated ESCs and/or iPSCs, including Klf family members, c-Myc and Lin28 [6], [7]. Understanding the exact role and mechanism of action of these and other regulators in ESC self-renewal is an important goal in developmental biology and will aid the practical use of PSCs.

Although ESCs from different species share the same key properties of pluripotency and self-renewal, major differences were found between murine (mESCs) and human ESCs (hESCs) including expression of different sets surface markers and distinct growth factor requirements [8]. Compared to mouse ESCs, hESCs display a characteristic flattened colony morphology, relatively slow growth and inefficient clonal propagation [3]. These properties resemble mouse epiblast-derived stem cells (EpiSC – referred to as “primed” hereafter), and indeed the gene expression profile of hESCs is closer to that of mouse EpiSC [9], [10]. Thus, current evidence suggests that hESCs are derived from a later developmental stage (primed) relative to the stage from which mouse ESCs are derived (naïve). Some progress has been made to push human ESCs toward the naïve state through genetic manipulation or by altering culture conditions [11], [12], but much work remains in order to unravel the differences between pluripotent state and species differences. While the “primed” model of hESCs might reconcile some of the differences between murine and human ESCs, it opens a fundamental question about the similarity of the transcriptional circuitry between the two ESC types.

Previously, we demonstrated a role for the transcription factor Zfx in the self-renewal of mESC and adult hematopoietic stem cells [13]. Zfx is encoded on the mammalian X chromosome and contains a transcription activation domain and a zinc finger domain for sequence-specific DNA binding. A highly homologous protein called Zfy is encoded on the Y chromosome and is expressed in human but not in murine male somatic cells. ZFX/ZFY genes are highly conserved in vertebrates, with ∼97% amino acid identity between murine and human ZFX in the DNA binding domain. The deletion of Zfx in mESC impairs self-renewal but does not affect differentiation capacity. Conversely, Zfx overexpression enhanced mESC self-renewal under suboptimal conditions and opposed both spontaneous and directed differentiation. Zfx directly activated functionally relevant mESC-specific target genes such as Tbx3 and Tcl1. Subsequent work has implicated Zfx in a common genetic pathway with Myc and Klf4, the two transcription factors controlling mESC self-renewal and iPSC reprogramming [14]–[16]. Thus, Zfx emerged as an essential and specific regulator of self-renewal in mESC, warranting the investigation of its role in the human system.

Here, we used a genetic approach to analyze the role of ZFX in hESC self-renewal and differentiation. Lentiviral shRNA knockdown of ZFX impaired self-renewal of hESCs. A novel bacterial artifical chromosome (BAC) transgenic strategy [17] was adapted to overexpress human ZFX in hESC under its native regulatory elements. Such gene dosage analysis showed that ZFX overexpression increased colony formation from single cells, a hallmark of improved hESC self-renewal. Furthermore, ZFX overexpression prevented spontaneous hESC differentiation under suboptimal conditions. Array analysis showed that ZFX overexpression decreased a number of genes characteristic of differentiated cells. These data establish an important role for ZFX in the regulation of hESC, confirming the conservation of self-renewal mechanisms in murine and human ESC.

## Materials and Methods

### ZFX Knockdown Human ESCs

H9 (WA-09) hESCs were maintained on MEFs (GlobalStem, Inc., Rockville, MD) before feeder-free expansion on Matrigel with conditioned media and 10 ng/ml FGF2 (3–5 days). To prepare cells for transduction, hESCs were exposed to Accutase (Innovative Cell Technologies, Inc., San Diego, CA) to create a single-celled suspension before plating at 18,000 cells/cm^2^ in the presence of 10 µM Y-27632. The following day, cells were transduced at a multiplicity of infection of 1 and 0.1. For each knockdown, phenotypes were observed at both multiplicities relative to the scrambled control. Puromycin selection began two days after transduction and no mock transduced cells remained two days after selection began. At this time, 5,000 puromycin resistant cells were replated per condition and were expanded in conditioned media +10 ng/ml FGF2 for seven days before crystal violet staining.

### Quantitative Immunofluoresce

Cells were transduced at a multiplicity of ∼0.1 as described above and puromycin selection was applied as described. Cells were fixed in 4% paraformaldehyde for 15 minutes before rinsing four times with PBS. Fixed cells were blocked/permeabilized with 1× Perm/wash buffer (BD Biosciences, San Jose, CA) for 20 minutes before exposure to ZFX primary antibody overnight at 4°C. The next day, cells were washed three times in Perm/wash buffer before exposure to secondary antibodies conjugated to Alexa 488 and Hoechst 33258. Cells were washed three times after the hour, and PBS was added to the cells for imaging.

Imaging was performed on an Operetta High Content Screening System. The Hoechst was exposed for 20 mSec and the Alexa 488 signal was captured by a 300 mSec exposure using 50% intensity from the light source. Harmony software version 3.0 was used to identify cell nuclei (Hoechst signal) and quantitated the Alexa 488 pixel intensity (ZFX protein) for each cell nucleus. The mean pixel intensity from 50 cell fields per sample were imaged from 3 independent experiments for quantitation. All knockdowns were significantly different from the scrambled control (p<0.0001 for pairwise t-tests). 11,803 (Scr), 10,296 (Z2), 4,765 (Z3) and 6,905 (Z4) nuclei were measured in these experiments.

### ZFX Overexpressing Human ESCs

Human ESCs were maintained as previously described [18]. BAC overexpressing human ESCs were made essentially as described for transgenic reporter BAC integration [17]. Briefly, H9 (WA-09) hESCs were maintained on MEFs (GlobalStem, Inc., Rockville, MD) before feeder-free expansion on Matrigel with conditioned media and 10 ng/ml FGF2 (3–5 days). To prepare cells for nucleofection, Accutase (Innovative Cell Technologies, Inc., San Diego, CA) was used to create a single-celled suspension, and 5 million cells were used per nucleofection (solution V, protocol B-16; Lonza, Cologne, Germany) with 3 ug of human Zfx BAC (RP11-1107D4) retrofitted with pRetroES [19]. Each nucleofection reaction was plated onto Neo-resistant MEFs (GlobalStem, Inc., Rockville, MD) in 10 µM Y-27632 for two days after nucleofection (Tocris Bioscience, Bristol, UK), and cells were allowed to recover for 4 days. Selection began with 25 ug/ml G418 (days 4–14; Invitrogen Life Science, Carlsbad, CA) before increasing selection to 40 ug/ml (days 14–20). Drug-resistant colonies were manually dissected before expansion into lines.

### Quantitative PCR Analysis

Real-time PCR analysis was performed using the following QuantiTect primers: POU5F1, CXCR4, PAX6, GAPDH and ACTN2. Other gene expression levels were determined by qPCR using the following primers:

ZFX

F: 5′-CGTAGGAGAGGAGGATGCTG-3′

R: 5′-TGCCTGGAATCAGGTCTTCT-3′

EOMES

F: 5′-CGGCCTCTGTGGCTCAAA-3′

R: 5′-AAGGAAACATGCGCCTGC-3′

ID2

F: 5′-AATCCTGCAGCACGTCATCG-3′

R: 5′- CTGGTGATGCAGGCTGACAA-3′

VIM

F: 5′-GGAGCTGCAGGAGCTGAATG-3′

R: 5′-GACTTGCCTTGGCCCTTGAG-3′

Septin 5

F: 5′-TCAACATCGTGCCTCTCATC-3′

R: 5′-GCTGCTTGAAGTCCTCATCC-3′

RNA was isolated using Trizol (Invitrogen, #15596-018) and reversed transcribed with the QuantiTect Reverse Transcription kit (Qiagen, #205313). SYBR green PCR reactions were performed using the PerfeCta SYBR Green SuperMix (Quanta Bioscience, #95054-500) on an Eppendorf Mastercycler Realplex^2^.

### Stem Cell Marker Expression

Direct immunofluorescence was performed using SSEA-4 Alexa Fluor® 488 (BD Pharmingen, #560308, La Jolla, CA), TRA1-81 Alexa Fluor® 555 (BD Pharmingen, #560123, La Jolla, CA), and Oct3/4 Alexa Fluor® 647 (BD Pharmingen, #560307, La Jolla, CA) according to the manufacturers recommendations.

### Western Blotting

Protein samples of H9 hESC and ZFX^Over^ hESC clones were prepared in RIPA buffer plus protease inhibitors (Roche Diagnostics Ltd.). Protein concentration was determined using Bradford assay and 30 ug or 15 ug of each sample were separated by 10% polyacrylamide gel. Gels were transferred to PVDF membranes using the Bio-Rad mini-gel transfer apparatus. Membranes were blocked with 3% milk for 1 hour at room temperature and probed with affinity-purified polyclonal anti-Zfx antibody (in 3% Milk, 1∶1000 overnight at 4°C). After washing 3X with TBST, membranes were next incubated with peroxidase-labeled goat anti-rabbit IgG (1∶10,000 in 3% milk) for 1 hour at room temperature before visualization with ECL.

### Spontaneous Differentiation Assay

Human ESCs were expanded on Matrigel-coated dishes in standard human ESC media containing 6 ng/ml FGF2 without conditioned media. After seven days, single-celled suspensions were made by dissociating cells with Accutase (Innovative Cell Technologies, Inc., San Diego, CA) for 45 minutes before being washed twice with human ESC media. Aliquots of the cells were stained with anti-SSEA-3 (SSEA-3 Alexa Fluor® 647; unpublished, BD Pharmingen, La Jolla, CA) and anti-SSEA-1 antibodies (SSEA-1 Alexa Fluor® 647; BD Pharmingen #560120, La Jolla, CA) before quantitation on a FACSAria. For this assay, a number of unrelated BAC transgenic cell lines were used as normal clone controls (see Table 1). These lines do not overexpress ZFX but have undergone the same clonal selection as the ZFX^Over^ clones (data not shown). Control clones were made from BACs with: ID1::YFP (clone 2) [20], HES5::GFP (clone 10) and DLL1::GFP (clones 281 and 277) [17]. All control clones were derived from H9 hESC and were made with the same nucleofection/selection protocol [17].

**Table 1:** All control hESC lines and clones used in this manuscript.

Figure	Control	Source
2	H9 and ZFX^Normal^	This manuscript
3	H9 and ZFX^Normal^	This manuscript
4	H9, DLL1::GFPc277 and c281, HES5::GFP c10 and ID1::YFPc2	Placantonakis et al., 2009 and James et al., 2010
5	H9, ID1::YFPc2 and DLL1::GFPc277	Placantonakis et al., 2009 and James et al., 2010
6	H9 and ZFX^Normal^	This manuscript
S1	H9 and ZFX^Normal^	This manuscript
S2	H9 and ZFX^Normal^	This manuscript
S3	H9 and ZFX^Normal^	This manuscript
S4	H9 and ZFX^Normal^	This manuscript
S5	H9, DLL1::GFPc277 and c281, HES5::GFP c10 and ID1::YFPc2	Placantonakis et al., 2009 and James et al., 2010

### Clonogenic Assay

For colony-forming cell assays, human ESC were dissociated with Accutase (Innovative Cell Technologies, Inc., San Diego, CA) for 45 minutes. Dissociated cells were washed twice with human ESC media. Serial dilutions of hESCs were plated onto Matrigel-coated 6 well dishes in conditioned media with 10 ng/ml FGF2. Cells were exposed to Y-27632 for 24 hours after plating, and colonies were visualized after 7 days by staining with crystal violet. The data presented are the number of colonies from the 1∶100 dilution and represent the number of colonies derived from 2,666 cells in 9.6 cm^2^.

Crystal violet staining was used because it is easier and cheaper and works as well for determining the number of colony forming units in a given culture. Figure S1A shows the alkaline phosphatase staining (Sigma, 86R-1KT) in the colony-forming assay: nearly every colony stains positive. We confirmed this result by growing clonal colonies before performing Oct4 FACS analysis (Figure S1B). Over 97.5% of the cells from all genotypes were Oct4+ demonstrating that these growth conditions strongly select for undifferentiated hESCs. This makes alkaline phosphatase detection a redundant feature of the colony growth assay.

### Directed Differentiation

To direct human ESCs to endoderm, we used a previously published protocol [21]. Briefly, endoderm induction was performed by switching from hESC media to RPMI with glutamine, 0.5% Hyclone FBS and 100 ng/ml Activin A. Cells were fed daily and were assayed on days 1 and 3. ESCs were directed to neural cells through co-culture with MS-5 bone marrow stromal cells as previously described [18].

### Gene Expression Array

RNA was isolated from the Tra1-81^HI^/SSEA-3^HI^ fraction of ZFX^Over1,2^, ZFX^Normal^ and H9 hESCs using Trizol (Invitrogen). Samples were labeled and hybridized to Illumina human 6 oligonucleotide arrays. Normalization and model-based expression measurements were performed using the Illumina analysis package (LUMI) available through open-source Bioconductor project (www.bioconductor.org) within the statistical programming language R (http://cran.r-project.org/). Pairwise comparisons were performed using the Linear Models for Microarray Data package (LIMMA) available through Bioconductor. Genes found to have an adjusted p-value <0.05 were considered significant.

### Statistical Analysis

Statistical analysis was performed using Prism for Mac version 5.0a. Unpaired, two-tailed T tests were performed comparing control cell lines versus ZFX^Over^ clones.

### BAC FISH

#### Metaphase harvest

Sub-confluent cultures were treated with 0.1 ug/ml Colcemid (Karyomax, Invitrogen) for 60–90 minutes before harvesting according to standard cytogenetics procedures. Briefly, cells were trypsinized to a single cell suspension, pelleted at 250 g for 5 minutes and resuspended in warm 0.075M KCl. After 8 minutes incubation at 37°C, the hypotonic was diluted with approximately 1/4 volume of 3∶1 methanol/glacial acetic acid fixative, gently mixed, and the cells pelleted as before. The supernatant was removed and the cell pellet loosened by gently flicking the base of the tube. The cells were then fixed in 3 changes of fixative. Fixed cell suspensions were stored at −20°C. Fixed metaphase preparations were dropped onto dry slides and the quality of spreading assessed by phase microscopy. Slides were then air-dried and aged at 37°C overnight.

#### Hybridization

DNA for human BAC clone RP11-1107D4 (BACPAC Resources, Children’s Hospital and Research Center at Oakland), spanning the ZFX locus, was labeled by nick translation with Red dUTP (Enzo Life Sciences Inc., supplied by Abbott Molecular Inc.), and hybridized to metaphase slides. Briefly, approximately 100 ng of labeled probe and 1 ug human Cot-1 DNA (Invitrogen) was mixed with hybridization buffer (50% formamide, 2×SSC,10% dextran sulfate, 0.1% SDS, 1×Denhardt’s solution, 40 mM sodium phosphate buffer, pH7) and applied to each slide before sealing under a coverslip with rubber cement. The slides were then placed in a HYBrite™ (Abbott Molecular), denatured at 72°C for 3°minutes, and then hybridized at 37°C overnight. After coverslip removal in 2×SCC, 0.1% Igepal CA 630 at room temperature, the slides were washed in 0.4×SSC, 0.3% Igepal at 73°C for 2°minutes, then in 2×SCC, 0.1% Igepal at RT, and rinsed briefly in 2×SSC. The slides were then stained in 0.08 µg/ml DAPI in 2×SSC for 3 minutes, rinsed, air-dried, then mounted in antifade solution (Vectashield, Vector Labs), and stored at 4°C. Slides were scanned using a Zeiss Axioplan 2i epifluorescence microscope equipped with a megapixel CCD camera (CV-M4^+^CL, JAI) controlled by Isis 5.2 imaging software (Metasystems Group Inc, Waltham, MA). At least 10 metaphases and 5 interphase nuclei were examined for each preparation.

## Results

### ZFX Knockdown in Human ESCs

**Figure 1:**
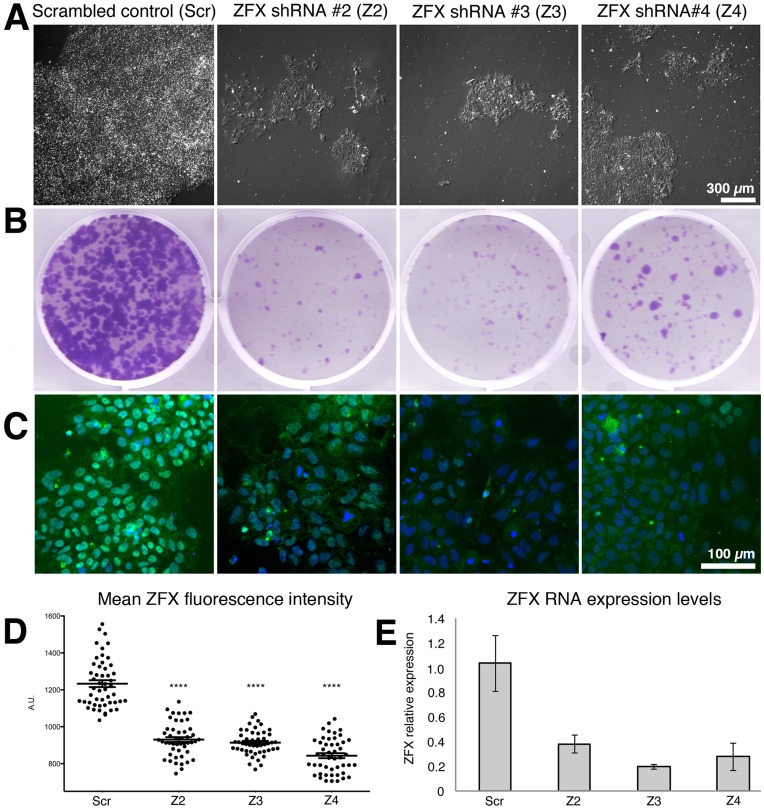
ZFX knockdown impairs hESC colony size. Human ESCs were transduced with ZFX knockdown lentiviral constructs and a scrambled control before clonal replating. (A) Live cell images and (B) and the entire well stained with crystal violet seven days after replating. (C) ZFX immunofluorescence after knockdown in hESCs, and (D) ZFX quantitative immunofluorescence analysis. Each dot is the average pixel intensity of nuclear ZFX protein averaged from all cells in one microscopic field. The average pixel intensity from 50 microscopic fields derived from 3 independent experiments is shown. The crosshairs and whiskers represent the mean and SEM. All knockdowns were significantly different from the scrambled control (p<0.0001 for t-tests of each knockdown versus scrambled). 11,803 (Scr), 10,296 (Z2), 4,765 (Z3) and 6,905 (Z4) nuclei were measured in these experiments. (E) ZFX RNA levels as measured by quantitative PCR after knockdown.

Zfx is essential for the self-renewal of mouse ESCs and hematopoietic stem cells. To test whether ZFX is required in human ESCs, we used a lentiviral vector system to efficiently and stably knockdown (KD) ZFX in hESCs. To characterize the extent of ZFX knockdown, initial experiments were performed in leukemia cell lines where three of the five shRNAs tested caused a strong knockdown of ZFX expression levels and a concomitant growth impairment (data not shown). H9 hESCs cells were transduced with these 3 ZFX knockdown viruses before puromycin selection. All 3 of the vetted ZFX knockdowns caused a marked decrease in the number and size of colonies relative to the scrambled shRNA control (Figure 1A and 1B). We confirmed that ZFX RNA (Figure 1C) and protein (Figure 1D) levels were reduced. This data suggests that ZFX expression is required for the efficient self-renewal of hESCs similar to its role in mESCs. We next turned to ZFX overexpression since the ZFX knockdown cells could not be readily expanded, confounding analysis.

### Production of ZFX Overexpressing Human ESCs

**Figure 2:**
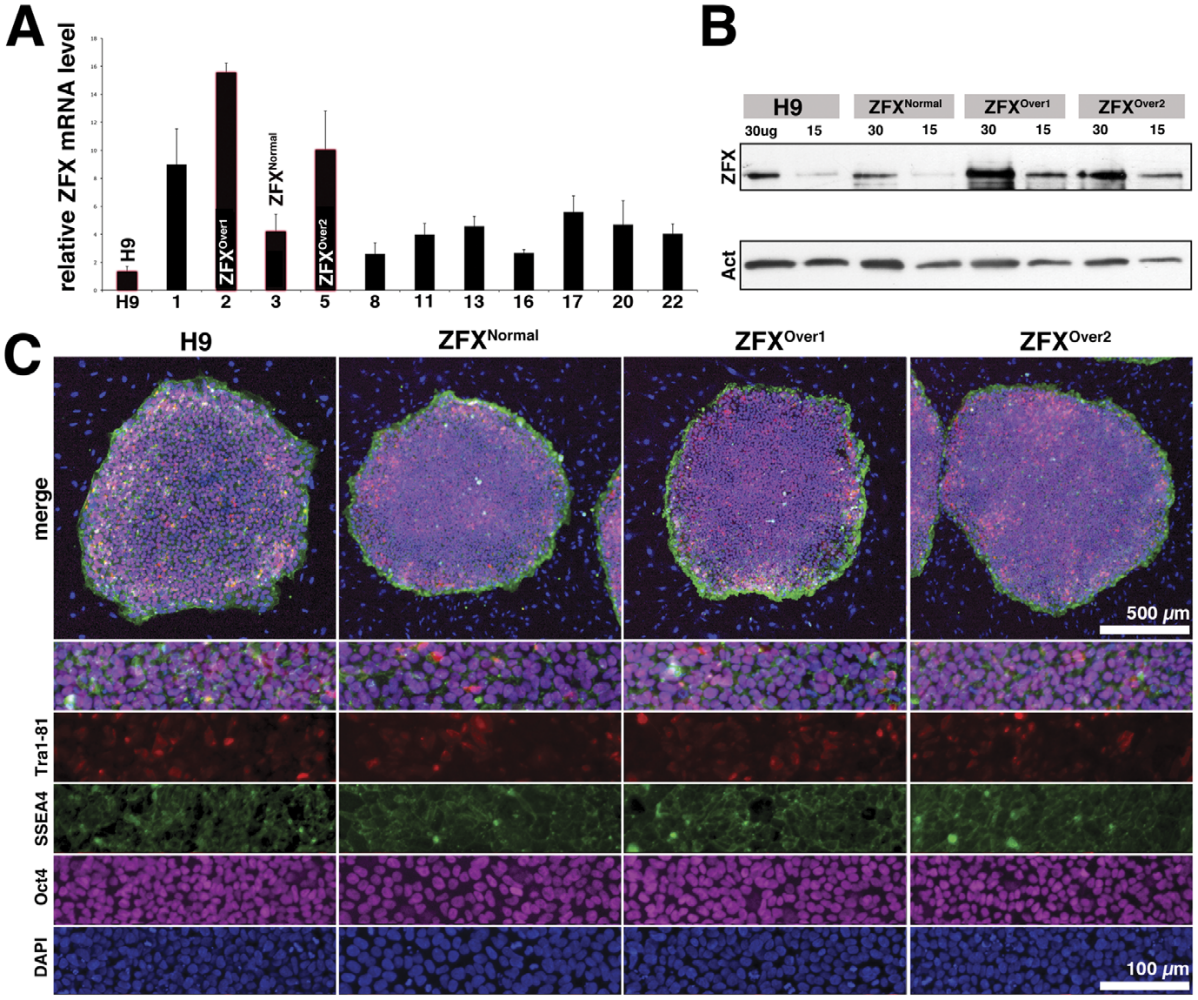
ZFX BAC transgenic human embryonic stem cells. A. Normalized *ZFX* expression levels from G418-resistant hESC clones with the original clone names shown on the x-axis. Three clones were renamed ZFX^Over1^, ZFX^Over2^ and ZFX^Normal^ were selected for further analysis. ZFX^Normal^ showed normal levels of ZFX but had undergone the same clonal and selection steps as ZFX^Over^ clones. B. ZFX and actin protein levels determined by Western blot analysis of ZFX^Over^ clones and controls. C. Oct4 (purple), SSEA-4 (green), Tra1-81 (red) and DAPI (blue) on ZFX^Over^ clones and controls. Scale bar  = 500 µm (low) or 100 µm on the high magnification images.

Similar to many transcription factors, the overexpression of ZFX cDNA from a heterologous promoter is toxic to cells (B.R., unpublished data). To overexpress murine Zfx under its native regulation, the entire genomic Zfx locus has been introduced into mESC as a bacterial artificial chromosome (BAC) transgene [13]. We have developed a method for stable BAC introduction into hESC to serve as reporter transgenes [17]. We adapted this approach to introduce additional copies of the human ZFX locus as BAC transgene in the H9 hESC. Several nucleofected H9 clones showed increased ZFX mRNA expression by quantitative RT-PCR (Figure 2A). We chose two clones (ZFX^Over1^ and ZFX^Over2^) with the highest ZFX expression levels and compared them to the parental H9 line, a control clone (ZFX^Normal^) with normal ZFX expression levels from the same experiment, as well as independent H9 GFP and YFP-expressing BAC transgenic reporter lines from our earlier studies (see Table 1 for a comprehensive description of controls used in this study) [17], [20]. We verified that the BAC reporter control clones expressed normal amounts of ZFX (data not shown).

As shown in Figure 2B, ZFX^Over^ clones expressed more ZFX protein than the parental hESC line (H9) or ZFX^Normal^ (Figure 2B). Fluorescent in situ hybridization (FISH) showed that the ZFX^Over^ clones harbored multiple copies of the BAC integrated at a single site while ZFX^Norma^l lacked detectable ZFX BAC DNA despite being drug resistant (Figure S2). Furthermore, ZFX^Over2^, the control clone and the H9 parental line had normal karyotypes, whereas ZFX^Over1^ showed a mixture of normal cells and karyotypically abnormal cells (data not shown). Stem cell surface markers on each clone looked similar to H9: they were positive for hESC markers SSEA-4+ and Oct-4+ and variegated for Tra1-81 (Figure 2C; Figure S3) suggesting that all clonal lines are in a similar undifferentiated state.

### ZFX-overexpressing hESC are More Clonogenic

**Figure 3:**
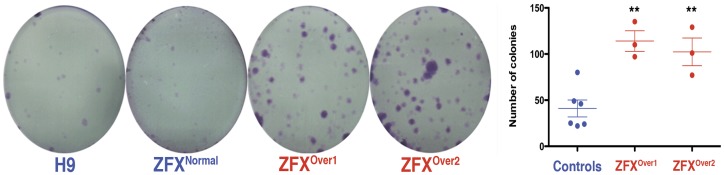
ZFX^Over^ cultures have a higher clonogenic capacity. A. ZFX^Over^ clones, ZFX^Normal^ and H9 hESCs (together grouped as controls) were dissociated into single cells before clonal replating. Cells were expanded for 10 days before fixation and staining with crystal violet. B. Colony counts between ZFX^Over^ and control hESCs in 3 independent experiments with error bars representing the S.E.M.

Under standard growth conditions, all hESC clones had similar morphologies, growth and apoptosis rates as the parental H9 hESC line (Figure S4). To quantitatively assess the self-renewal capacity of ZFX^Over^ versus ZFX^Normal^ and H9 hESCs at the undifferentiated stage, we analyzed their growth behavior following plating cells at clonal densities. The mean number of colonies formed from ZFX^Over^ clones was significantly higher than controls (114.00±11.15 for ZFX^Over1^ and 102.3±15.03 for ZFX^Over2^ compared to 41.00±9.16 for H9 and ZFX^Normal^. p = 0.0020 for ZFX^Over1^ and 0.0078 for ZFX^Over2^). In addition to colony number, the size of the ZFX^Over^ colonies derived from single cells was larger compared to controls (Figure 3 and Figure S1). These data demonstrate that ZFX overexpression in hESCs results in cultures enriched in colony-forming cells, a sensitive assay of the undifferentiated state [22].

### Reduced Spontaneous Differentiation in ZFX^Over^ cells

**Figure 4:**
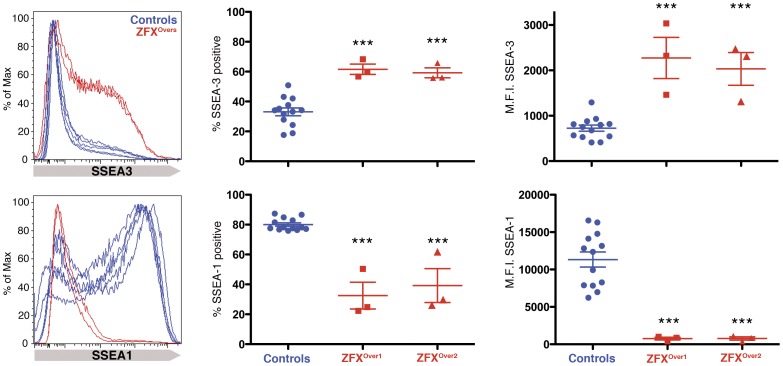
ZFX^Over^ clones resist spontaneous differentiation. A. ZFX^Over^ clones and controls were expanded in conditions promoting self-renewal before SSEA-3 and SSEA-1 FACS analysis. B. ZFX^Over^ clones and controls were expanded in suboptimal conditions before SSEA-3 and SSEA-1 FACS analysis. The quantitation compares ZFX^Over^ clones to controls in three independent experiments and error bars represent the S.E.M. See Figure S5 for all 3 independent experiments.

Next we tested the growth of ZFX^Over^ clones under conditions suboptimal for hESC self-renewal. ZFX^Over^ and control hESC lines were cultured in the absence of conditioned media or feeder cells to promote spontaneous differentiation, and the percentage of cells expressing SSEA-3 and SSEA-1 was measured to quantify the ratio of undifferentiated versus differentiated cells, respectively. In control experiments, there was no statistical difference in marker expression when conditioned media was used to expand cells without feeders (data not shown). However, in suboptimal conditions, H9 and the control clones showed strong signs of differentiation while the ZFX^Over^ cells resisted spontaneous differentiation (Figure 4; Figure S5). The control cultures had low percentages of the undifferentiated hESC marker SSEA-3 (combined mean of 33.12±2.62% SEM; n = 13) while ZFX^Over1^ (61.57±3.48% SEM, n = 3; p = 0.0002) and ZFX^Over2^ (59.20±3.30% SEM, n = 3; p = 0.0005) maintained significantly higher levels of SSEA-3. Similarly, the mean fluorescence intensity was significantly higher in ZFX^Over1^ (2273±454.7 arbitrary units, or AU SEM, n = 3; p<0.0001) and ZFX^Over2^ (2033±362.3 AU SEM, n = 3; p<0.0001) compared with the pooled controls (724.9±67.21 AU SEM, n = 13).

Reciprocally, H9 and control clones expressed high levels of SSEA-1, a marker of differentiated cells (combined mean 80.05±1.14% SEM, n = 13; p<0.0001) compared to ZFX^Over1^ (32.50±8.98 AU SEM, n = 3; p<0.0001) and ZFX^Over2^ (39.23±11.33 AU SEM, n = 3; p<0.0001). The mean fluorescent intensity of SSEA-1 expression was also significantly higher in controls (11,326±1008 AU SEM, n = 13) compared to ZFX^Over1^ (760.7±188 AU SEM, n = 3; p = 0.002) and ZFX^Over2^ (772.7±211.2 AU SEM, n = 3; p = 0.0002). Taken together, these data show that ZFX overexpression promotes self-renewal of hESCs and inhibits differentiation in suboptimal culture conditions.

**Figure 5:**
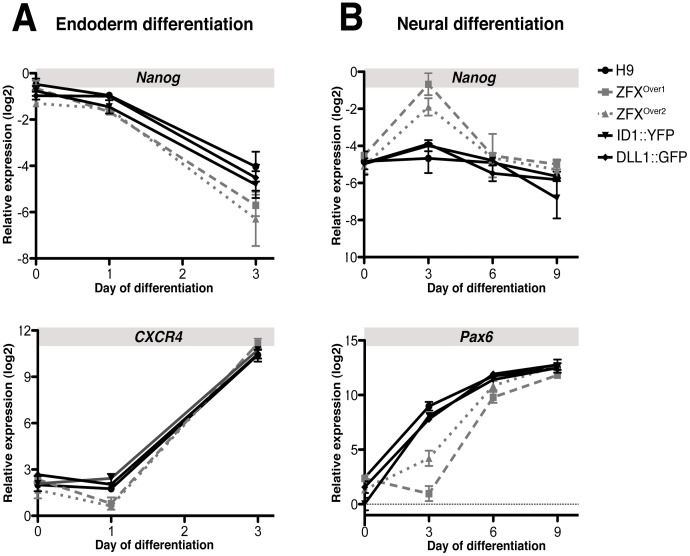
Directed differentiation of ZFX^Over^ clones to endoderm and neural tissue. ZFX^Over^ clones, two control clones that express normal ZFX levels (ID1::YFPc2, Dll1::GFPc277) and H9 were directed to endoderm or neural cells, and the level of Nanog, Pax6 (neural) and CXCR4 (endoderm) mRNA at each time point was measured by quantitative PCR.

To test whether higher ZFX levels globally prohibit ESC differentiation, ZFX^Over^ clones, two BAC transgenic control clones (ID1::YFPc2 and Dll1::GFPc277) and H9 were directed into endoderm or neural tissue and we examined lineage marker expression during differentiation using quantitative RT-PCR. We detected no significant difference in the loss of Nanog expression or gain in CXCR4 expression in ZFX^Over^ clones during endodermal differentiation (Figure 5). During neural differentiation, ZFX^Over^ clones showed delayed Nanog downregulation and Pax6 induction on day 3 but no difference at later time points. These data show that ZFX overexpression reduces spontaneous differentiation yet permits directed differentiation, thus leaving hESC pluripotency intact.

### Gene Expression Profile of ZFX^Over^ cells

**Figure 6:**
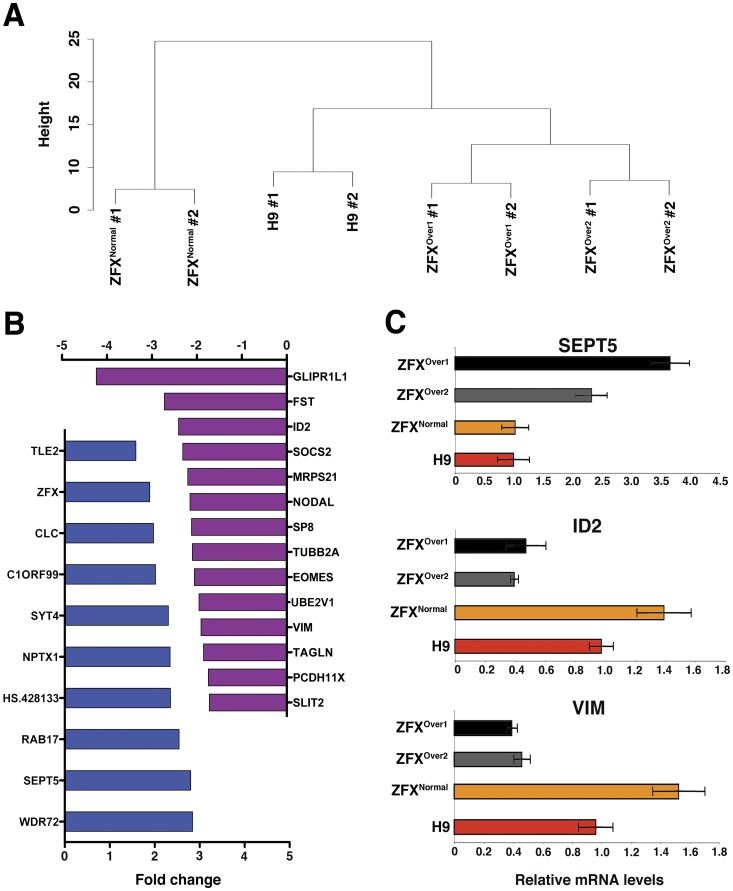
Gene expression analysis of ZFX^Over^ clones compared to controls. Two independent samples of Tra1-81^HI^/SSEA-3^HI^ hESCs were isolated from ZFX^Over^ clones, ZFX^Normal^ and H9 hESC array analysis. A. Dendrograms of each cell line after clustering analysis. B. Up- and down-regulated genes in ZFX^Over^ compared to H9 using an adjusted p-value of 0.05 as a cutoff. C. Quantitative PCR validation of selected genes on the array.

To gain insight into the genome-wide expression changes due to ZFX overexpression, we performed microarray analysis in ZFX^Over^ hESCs. In order to avoid any systematic bias in the gene expression analysis due to varying levels of spontaneous differentiation in ZFX^Over^ versus controls, we isolated the most undifferentiated (Tra1-81^HI^/SSEA-3^HI^) hESCs from each line. Hierarchical clustering analysis of the microarray data (Figure 6A) showed that the ZFX^Normal^ clone was most distinct from other hESC, possibly as a result of nucleofection/selection procedure. Nevertheless, principal component analysis identified genes changing in the two ZFX^Over^ clones compared to both ZFX^Normal^ and the parental H9 cells. Similar results were obtained when ZFX^Normal^ was excluded from the analysis (Figure 6B). The genes overexpressed in ZFX^Over^ cells included *ZFX* itself, thus validating the analysis (Figure 6B). The differential expression of select genes was confirmed by qPCR on independent samples (Figure 6C). Notably, many genes reduced in ZFX^Over^ cells represent canonical markers of differentiation, such as NODAL, EOMES and VIMENTIN. Thus, ZFX^Over^ hESC show reduced baseline expression of lineage differentiation markers, compatible with the hypothesis that ZFX overexpression stabilizes the undifferentiated state of hESC.

## Discussion

In the current study we demonstrate a role for the transcription factor ZFX in modulating the self-renewal of hESC using gain- and loss-of-function approaches. ZFX reduction caused a loss of self-renewal while BAC-mediated ZFX overexpression increased the clonogenicity and decreased spontaneous differentiation of hESCs. Importantly, ZFX-overexpressing clones retained their ability to undergo differentiation in response to appropriate stimuli. The use of BAC transgenesis was critical to circumvent general toxic effects of ZFX overexpression observed using heterologous promoters. Gene expression driven by the endogenous gene locus in a BAC provides advantages over heterologous promoters, such as native gene regulation, reduced position effect [23] and copy number-dependent expression [24]. Until now, BACs were used to direct reporter gene expression [17] or as vehicles for homologous recombination in hESC [25]. We believe this study is the first to demonstrate their utility as vectors for functional gene expression in hESCs.

Because the extrinsic self-renewal signals, morphology and clonogenicity differ between human and mouse ESCs, it is critical to identify the self-renewal regulators that are conserved between the two species. Here we provide evidence for the functional conservation of ZFX, a critical member of the self-renewal transcriptional network. Our gain-of-function studies in hESCs are compatible with our previous work in mESCs demonstrating enhanced self-renewal and reduced spontaneous differentiation in both murine and human ESC. However, ZFX-overexpressing hESC underwent normal lineage-specific differentiation *in vitro*, while forced differentiation in Zfx-overexpressing murine ESC led to a severely impaired differentiation response [13]. While we currently do not know the reason for these differences between mouse and human ESCs, we cannot rule out that they simply reflect different levels of overexpression (∼3-fold overexpression in hESC compared to >8-fold in the examined clones of murine ESC). Interestingly, the genes affected by Zfx overexpression in murine ESC (S.H., unpublished) and human ESC (this study) show little overlap. This may reflect the differences in the experimental approaches and microarray platforms used, and/or highlight the species-specific gene expression profiles of undifferentiated ESC. In any case, our data highlight the conserved cell-intrinsic molecular control of ESC self-renewal by ZFX, despite the differences in extrinsic signals.

The enhanced self-renewal observed in ZFX-overexpressing clones could reflect a reduction in the baseline heterogeneity of cultured ESC. This hypothesis is supported by the increased plating efficiency of ZFX^Over^ clones and by a small but reproducible increase in the percentage of cells expressing undifferentiated hESC markers (data not shown). It is possible that high ZFX levels stabilize a chromatin conformation that favors self-renewal over differentiation. This ‘locked’ state could also explain the kinetic delay in neural differentiation observed in ZFX-overexpressing hESC. Alternatively, ZFX overexpression may convert hESC from their primed state into a less differentiated, naïve state characteristic of murine ESC. Indeed, many of the genes downregulated in ZFX^Over^ clones are expressed in mouse epiblast stem cells isolated from the postimplantation embryo [9], [10]. However, clonal replating experiments without the ROCK inhibitor Y-27632 showed poor clonal replating despite ZFX overexpression (data not shown), arguing against a truly naïve state. Nevertheless, it is possible that pluripotency is a continuum between the naïve and primed states, and that ZFX overexpression has moved the primed human ESCs toward a naïve state. If true, our data on ZFX overexpression may aid in defining a currently elusive, mouse ESC-like, pluripotent state in hESCs. The functional characterization of the potential ZFX target genes in hESCs reported here may contribute to the identification of key evolutionarily conserved components of the ESC self-renewal machinery.

## Supporting Information

Figure S1 **Nearly all cells that survive the colony-forming assay are pluripotent.** A. Alkaline phosphatase staining of the clonal colony-forming assay. B. Cells were grown clonally in the colony forming assay before single cell dissociation, fixation, permeabilization and staining with Oct4-Alexa647 before FACS analysis.
(TIF)

Figure S2 **ZFX BAC transgenic human embryonic stem cells characterization.** Full images from Figure 2, showing Oct4 (purple), SSEA-4 (green) Tra1-81 (red) and DAPI (blue) on ZFX^Over^ clones and controls. Scale bar  = 100 µm.
(TIF)

Figure S3 **ZFX BAC FISH analysis identifies multiple copies of the BAC in each ZFX^Over^ clone at a single integration site.** ZFX^Normal^ and H9 show only endogenous ZFX whereas both ZFX^Over^ clones show endogenous (green arrows) an additional brighter spot (red arrows) revealing the ZFX BAC integration.
(TIF)

Figure S4 **ZFX^Over^ clones do not have differences in cell growth nor apoptosis during normal passage.** A. hESC lines were passaged on day 0 using dispase and were counted the day after passage to assess seeding (day 1). Six days later, cells were counted to assess growth kinetics (day 7). On day 7, cells were passaged again using dispase and counted the day after (day 8). The same process was repeated on days 14 and 15. B. hESC lines were grown in feeder free conditions and the number of cells with AnnexinV staining were quantitated. No differences were observed after dispase passage. Three independent experiments were performed both for A and B and the data were not significantly different in either case.
(TIF)

Figure S5 **All data from the spontaneous differentiation experiment shown in** **Figure 4** **.** Three independent experiments showing the level of SSEA-1 and SSEA-3 after 7 days of suboptimal culture. The mean fluorescent intensity is diagrammed on the right side of the figure: controls are grouped on the left side, ZFX^Over1^ in the middle and ZFX^Over2^ on the right.
(TIF)

## References

[1] Evans MJ, Kaufman MH (1981) Establishment in culture of pluripotential cells from mouse embryos. Nature 292: 154–156. 7242681 10.1038/292154a0

[2] Martin GR (1981) Isolation of a pluripotent cell line from early mouse embryos cultured in medium conditioned by teratocarcinoma stem cells. Proc Natl Acad Sci U S A 78: 7634–7638. 6950406 10.1073/pnas.78.12.7634 PMC349323

[3] Thomson JA, Itskovitz-Eldor J, Shapiro SS, Waknitz MA, Swiergiel JJ, et al (1998) Embryonic stem cell lines derived from human blastocysts. Science 282: 1145–1147. 9804556 10.1126/science.282.5391.1145

[4] Chambers I, Tomlinson SR (2009) The transcriptional foundation of pluripotency. Development 136: 2311–2322. 19542351 10.1242/dev.024398 PMC2729344

[5] Do JT, Scholer HR (2009) Regulatory circuits underlying pluripotency and reprogramming. Trends Pharmacol Sci 30: 296–302. 19427042 10.1016/j.tips.2009.03.003

[6] Takahashi K, Tanabe K, Ohnuki M, Narita M, Ichisaka T, et al (2007) Induction of pluripotent stem cells from adult human fibroblasts by defined factors. Cell 131: 861–872. 18035408 10.1016/j.cell.2007.11.019

[7] Yu J, Vodyanik MA, Smuga-Otto K, Antosiewicz-Bourget J, Frane JL, et al (2007) Induced pluripotent stem cell lines derived from human somatic cells. Science 318: 1917–1920. 18029452 10.1126/science.1151526

[8] Rossant J (2007) Stem cells and lineage development in the mammalian blastocyst. Reprod Fertil Dev 19: 111–118. 17389140 10.1071/rd06125

[9] Brons IG, Smithers LE, Trotter MW, Rugg-Gunn P, Sun B, et al (2007) Derivation of pluripotent epiblast stem cells from mammalian embryos. Nature 448: 191–195. 17597762 10.1038/nature05950

[10] Tesar PJ, Chenoweth JG, Brook FA, Davies TJ, Evans EP, et al (2007) New cell lines from mouse epiblast share defining features with human embryonic stem cells. Nature 448: 196–199. 17597760 10.1038/nature05972

[11] Hanna J, Cheng AW, Saha K, Kim J, Lengner CJ, et al (2010) Human embryonic stem cells with biological and epigenetic characteristics similar to those of mouse ESCs. Proc Natl Acad Sci U S A 107: 9222–9227. 20442331 10.1073/pnas.1004584107 PMC2889088

[12] Xu Y, Zhu X, Hahm HS, Wei W, Hao E, et al (2010) Revealing a core signaling regulatory mechanism for pluripotent stem cell survival and self-renewal by small molecules. Proc Natl Acad Sci U S A 107: 8129–8134. 20406903 10.1073/pnas.1002024107 PMC2889586

[13] Galan-Caridad JM, Harel S, Arenzana TL, Hou ZE, Doetsch FK, et al (2007) Zfx controls the self-renewal of embryonic and hematopoietic stem cells. Cell 129: 345–357. 17448993 10.1016/j.cell.2007.03.014 PMC1899089

[14] Chen X, Xu H, Yuan P, Fang F, Huss M, et al (2008) Integration of external signaling pathways with the core transcriptional network in embryonic stem cells. Cell 133: 1106–1117. 18555785 10.1016/j.cell.2008.04.043

[15] Hu G, Kim J, Xu Q, Leng Y, Orkin SH, et al (2009) A genome-wide RNAi screen identifies a new transcriptional module required for self-renewal. Genes Dev 23: 837–848. 19339689 10.1101/gad.1769609 PMC2666338

[16] Ouyang Z, Zhou Q, Wong WH (2009) ChIP-Seq of transcription factors predicts absolute and differential gene expression in embryonic stem cells. Proc Natl Acad Sci U S A 106: 21521–21526. 19995984 10.1073/pnas.0904863106 PMC2789751

[17] Placantonakis DG, Tomishima MJ, Lafaille F, Desbordes SC, Jia F, et al (2009) BAC transgenesis in human embryonic stem cells as a novel tool to define the human neural lineage. Stem Cells 27: 521–532. 19074416 10.1634/stemcells.2008-0884

[18] Perrier AL, Tabar V, Barberi T, Rubio ME, Bruses J, et al (2004) Derivation of midbrain dopamine neurons from human embryonic stem cells. Proc Natl Acad Sci U S A 101: 12543–12548. 15310843 10.1073/pnas.0404700101 PMC515094

[19] Wang Z, Engler P, Longacre A, Storb U (2001) An efficient method for high-fidelity BAC/PAC retrofitting with a selectable marker for mammalian cell transfection. Genome Res 11: 137–142. 11156622 10.1101/gr.159001 PMC311050

[20] James D, Nam HS, Seandel M, Nolan D, Janovitz T, et al (2010) Expansion and maintenance of human embryonic stem cell-derived endothelial cells by TGFbeta inhibition is Id1 dependent. Nat Biotechnol 28: 161–166. 20081865 10.1038/nbt.1605 PMC2931334

[21] D’Amour KA, Agulnick AD, Eliazer S, Kelly OG, Kroon E, et al (2005) Efficient differentiation of human embryonic stem cells to definitive endoderm. Nat Biotechnol 23: 1534–1541. 16258519 10.1038/nbt1163

[22] O’Connor MD, Kardel MD, Iosfina I, Youssef D, Lu M, et al (2008) Alkaline phosphatase-positive colony formation is a sensitive, specific, and quantitative indicator of undifferentiated human embryonic stem cells. Stem Cells 26: 1109–1116. 18276800 10.1634/stemcells.2007-0801

[23] Lee EC, Yu D, Martinez de Velasco J, Tessarollo L, Swing DA, et al (2001) A highly efficient Escherichia coli-based chromosome engineering system adapted for recombinogenic targeting and subcloning of BAC DNA. Genomics 73: 56–65. 11352566 10.1006/geno.2000.6451

[24] Yang XW, Wynder C, Doughty ML, Heintz N (1999) BAC-mediated gene-dosage analysis reveals a role for Zipro1 (Ru49/Zfp38) in progenitor cell proliferation in cerebellum and skin. Nat Genet 22: 327–335. 10431235 10.1038/11896

[25] Song H, Chung SK, Xu Y (2010) Modeling disease in human ESCs using an efficient BAC-based homologous recombination system. Cell Stem Cell 6: 80–89. 20074536 10.1016/j.stem.2009.11.016

