# Supplementary figures and images for: ZFX Controls the Self-Renewal of Human Embryonic Stem Cells
Source: PLoS One. 2012 Aug 3;7(8):e42302. doi: 10.1371/journal.pone.0042302 (PMC3411758; doi:10.1371/journal.pone.0042302)

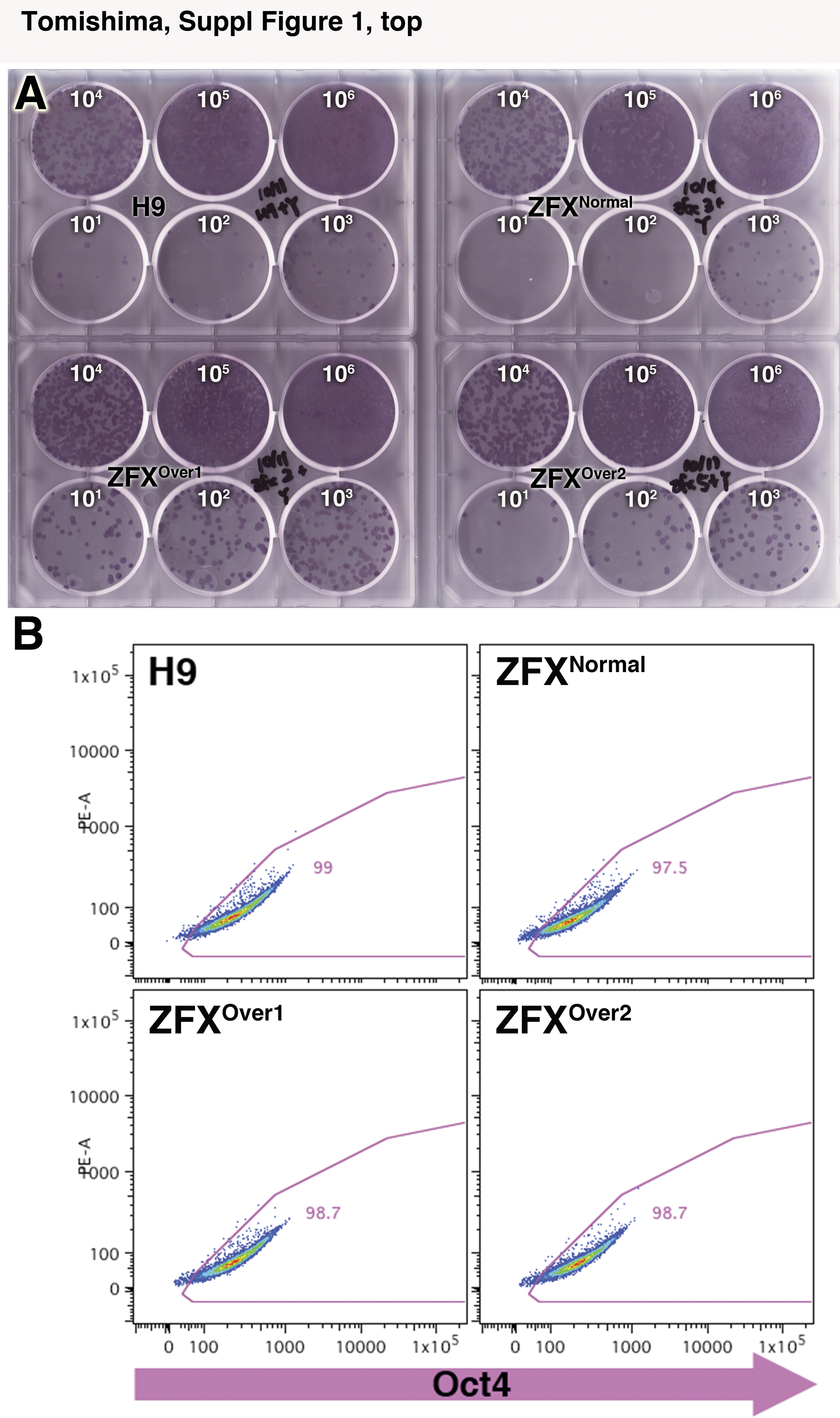

Supplement: Figure S1 — Nearly all cells that survive the colony-forming assay are pluripotent. A. Alkaline phosphatase staining of the clonal colony-forming assay. B. Cells were grown clonally in the colony forming assay before single cell dissociation, fixation, permeabilization and staining with Oct4-Alexa647 before FACS analysis. (TIF) [file pone.0042302.s001.tif]

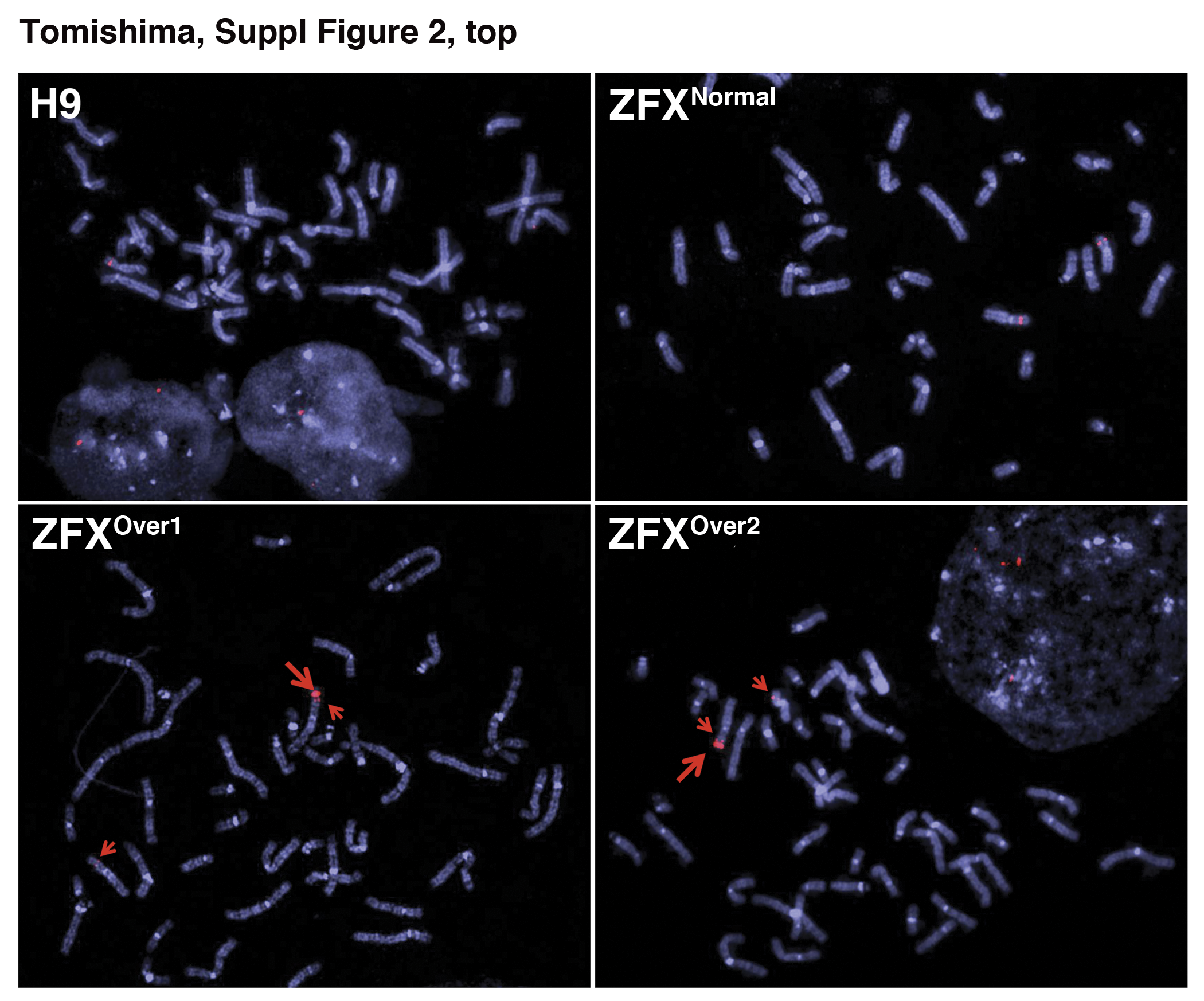

Supplement: Figure S2 — ZFX BAC transgenic human embryonic stem cells characterization. Full images from Figure 2, showing Oct4 (purple), SSEA-4 (green) Tra1-81 (red) and DAPI (blue) on ZFXOver clones and controls. Scale bar = 100 µm. (TIF) [file pone.0042302.s002.tif]

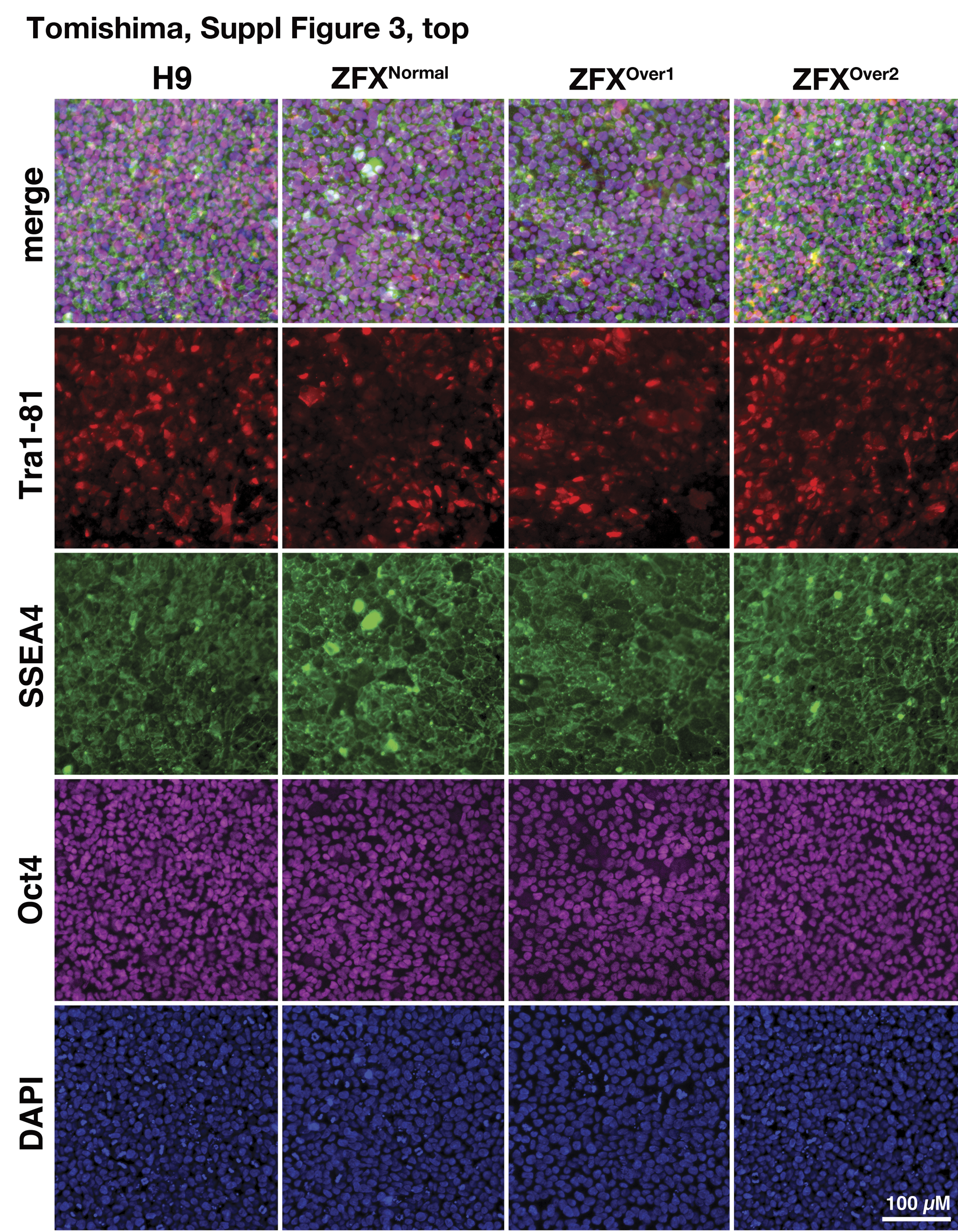

Supplement: Figure S3 — ZFX BAC FISH analysis identifies multiple copies of the BAC in each ZFXOver clone at a single integration site. ZFXNormal and H9 show only endogenous ZFX whereas both ZFXOver clones show endogenous (green arrows) an additional brighter spot (red arrows) revealing the ZFX BAC integration. (TIF) [file pone.0042302.s003.tif]

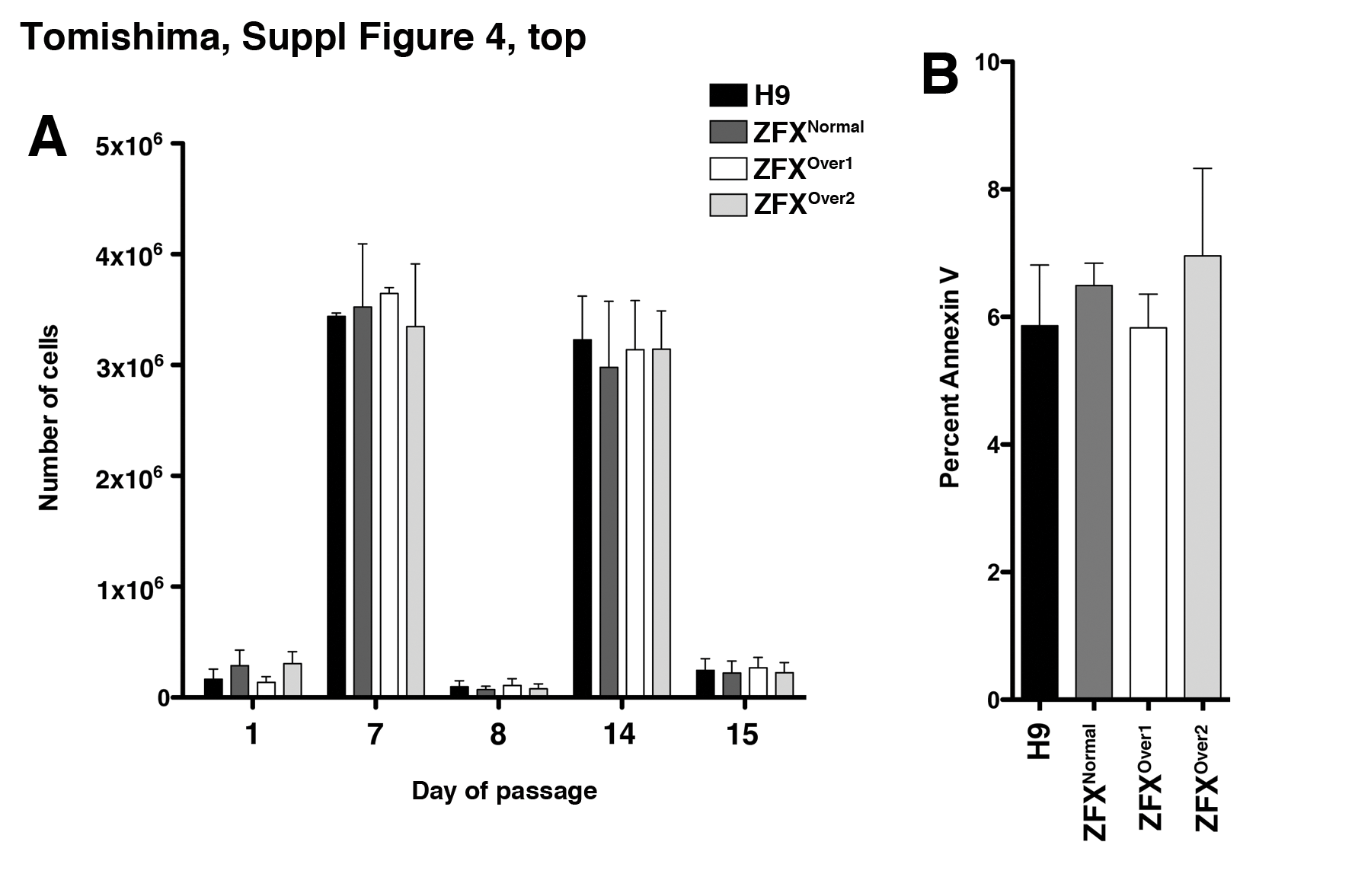

Supplement: Figure S4 — ZFXOver clones do not have differences in cell growth nor apoptosis during normal passage. A. hESC lines were passaged on day 0 using dispase and were counted the day after passage to assess seeding (day 1). Six days later, cells were counted to assess growth kinetics (day 7). On day 7, cells were passaged again using dispase and counted the day after (day 8). The same process was repeated on days 14 and 15. B. hESC lines were grown in feeder free conditions and the number of cells with AnnexinV staining were quantitated. No differences were observed after dispase passage. Three independent experiments were performed both for A and B and the data were not significantly different in either case. (TIF) [file pone.0042302.s004.tif]

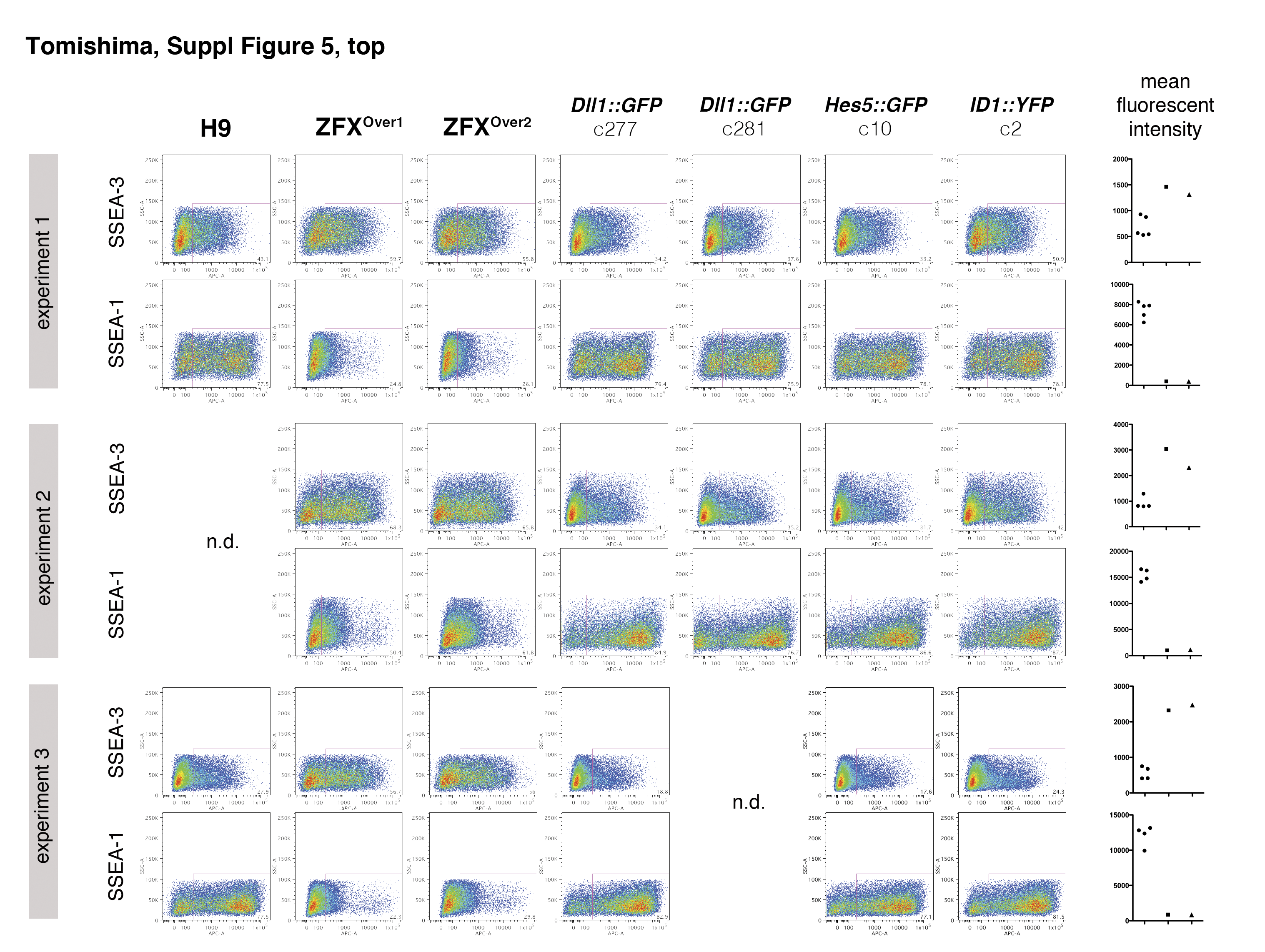

Supplement: Figure S5 — All data from the spontaneous differentiation experiment shown in Figure 4 . Three independent experiments showing the level of SSEA-1 and SSEA-3 after 7 days of suboptimal culture. The mean fluorescent intensity is diagrammed on the right side of the figure: controls are grouped on the left side, ZFXOver1 in the middle and ZFXOver2 on the right. (TIF) [file pone.0042302.s005.tif]
